# Effects of Packaging Materials on Structural and Simulated Digestive Characteristics of Walnut Protein during Accelerated Storage

**DOI:** 10.3390/foods12030620

**Published:** 2023-02-01

**Authors:** Miaomiao Han, Jinjin Zhao, Qingzhi Wu, Xiaoying Mao, Jian Zhang

**Affiliations:** School of Food Science and Technology, Shihezi University, Shihezi 832003, China

**Keywords:** walnut kernels, packaging materials, accelerated storage, protein oxidation, structural characterization, digestive properties

## Abstract

Walnuts are rich in fat and proteins that become oxidized during the processing and storage conditions of their kernels. In this study, the effect of three packaging materials (e.g., polyethylene sealed packaging, polyamide/polyethylene vacuum packaging, and polyethylene terephthalate/aluminum foil/polyethylene vacuum packaging) were investigated on the oxidation, structural and digestive properties of walnut kernel proteins. Results showed that the amino acid content gradually decreased and carbonyl derivatives and dityrosine were formed during storage. The protein molecule structure became disordered as the α-helix decreased and the random coil increased. The endogenous fluorescence intensity decreased and the maximum fluorescence value was blue-shifted. After 15 days of storage, surface hydrophobicity decreased, while SDS-PAGE and HPLC indicated the formation of large protein aggregates, leading to a reduction in solubility. By simulating gastrointestinal digestion, we found that oxidation adversely affected the digestive properties of walnut protein isolate and protein digestibility was best for polyethylene terephthalate/aluminum foil/polyethylene vacuum packaging. The degree of protein oxidation in walnuts increased during storage, which showed that except for fat oxidation, the effect of protein oxidation on quality should be considered. The results of the study provided new ideas and methods for walnut quality control.

## 1. Introduction

Walnut (*Juglans Regia* L.) belongs to the genus Juglans in the family Juglandaceae [1]. It is mainly distributed in Europe, North America, and Asia. Currently, China ranks first in the world in terms of walnut cultivation area and production [2]. Xinjiang is one of the main producing areas in China, with more than 391.13 thousand hectares and 1.15 million tons of walnut cultivation and production respectively in 2020, ranking second in China (FAOSTAT, http://faostat.fao.org/faostat/, accessed on 13 September 2022). Xinjiang walnuts are popular among consumers because of their unique ecological environment, characterized by thin shells and high unsaturated fatty acid content with excellent quality [3]. High intake of polyunsaturated fatty acids has the ability to strengthen the body’s immune system, fight cardiovascular disease and defend against the risk of neurological diseases [4]. However, the high content of unsaturated fatty acids easily caused oxidative rancidity of walnuts and affected their quality. Therefore, quality control of walnut storage has become an important issue that needs to be urgently addressed in the walnut industry.

Storage environment and food packaging are often involved in food storage. Walnut quality can be affected by oxygen, temperature, humidity, and natural light in the storage environment. Oxygen concentration is one of the most important environmental factors, and lipid oxidation can be inhibited by using low-oxygen permeability packaging materials or storing them in an environment with low-oxygen content [5]. Temperature is another important environmental factor affecting the quality of walnuts, followed by light conditions [6]. Christopoulos et al. [7] reported that walnuts stored in the dark at 5 °C for 25 weeks showed no rancid taste. These studies showed that environmental factors could lead to lipid oxidation and quality deterioration of walnuts, thus reducing their edible value and nutritional quality. Therefore, it is particularly important to use different types of methods to maintain walnut quality and reduce the influence of external factors on food quality during storage. Particularly, packaging materials with high water resistance, high air barrier, and light-blocking properties play an important role in controlling food quality.

Previous studies on the storage quality of walnuts have mainly focused on the effects of storage conditions on lipid oxidation and walnut quality [8,9,10], while few studies have involved changes in walnut proteins. However, the free fatty acids produced by lipid rancidity are extremely unstable and are prone to be oxidized further to lipid radicals and lipid secondary oxidation products, which indirectly induce protein oxidation and structural modifications, thus affecting the nutritional value and digestive properties of walnut proteins [11]. Wu et al. [12] found that rice rancidity products induced easily protein oxidation, leading to secondary structure disruption of protein, formation of aggregates, and cross-linking. Currently, research on protein oxidation has focused on the use of in vitro simulated oxidation systems to oxidize proteins and to evaluate the structural and functional properties of proteins, which revealed that free radicals and lipid oxidation products could indeed affect the structural and functional properties of proteins. Treatment of seeded watermelon kernel protein isolate (WMP) with 2′-Azobis (2-amidinopropane) dihydrochloride (AAPH) revealed a significant effect of AAPH on the structural and functional properties of WMP [13]. The ·OH oxidation led to the carbonylation of amino acid side chains, secondary structure α-helix conversion to β-sheet, tryptophan residues destruction, and aggregation of myofibrillar protein [14]. The action of reactive oxygen species (ROS) or lipid secondary oxidation products on proteins led to covalent protein modifications, including side chain group oxidation, backbone cleavage, unfolding, degradation, and aggregation [15]. 

At present, there are few studies on the oxidation and digestive properties of walnut proteins in storage. Therefore, in this experiment, walnut kernels were used as raw material and stored at high temperature and humidity conditions (40 °C, 70% relative humidity) for accelerated storage. The effects of different packaging materials (polyethylene sealed packaging, polyamide/polyethylene vacuum packaging, and polyethylene terephthalate/aluminum foil/polyethylene vacuum packaging) on protein oxidation, structural properties, and in vitro digestibility of walnuts were investigated. Pearson correlation analysis was used to reveal the relationship between each oxidation index, structural properties, and in vitro digestibility of walnuts during storage. The aim was to investigate the mechanism of protein quality changes during walnut storage, in order to provide a basis and reference for nutritional and quality control in walnut storage.

## 2. Materials and Methods

### 2.1. Materials

Walnut samples were purchased from a local agricultural market (Shihezi, Xinjiang Province, China). Then, walnuts were packed in polyethylene sealed packaging (PE: 20 × 28 cm, 0.1 mm; transparency); polyamide/polyethylene vacuum packaging (PA/PE: 20 × 28 cm, 0.18 mm; transparency) and polyethylene terephthalate/aluminum foil/polyethylene vacuum packaging (PET/AL/PE: 20 × 28 cm, 0.16 mm; non-transparent). The vacuum packaging was carried out by means of a vacuum packaging machine from Longao (Guangxi, China). Each package contained 500 g of walnut kernels for storage and each storage time was carried in triplicate. The samples were stored in constant temperature and humidity equipment (40 °C, 70% relative humidity) for 60 days to accelerate storage. All walnut samples were analyzed in triplicate at 0, 15, 30, 45, and 60 days. 

2,4-Dinitrophenylhydrazene (DNPH) and 8-Anilino-1-naphthalenesulfonic acid (ANS) were obtained from Yuanye (Shanghai, China). Pepsin (3000 μ/mg) and trypsin (250 μ/mg) were acquired from Boao (Shanghai, China). All other chemicals were of analytical reagent grade and were produced in China.

### 2.2. Preparation of Walnut Protein Isolate (WPI)

The WPI was prepared by the method of alkaline extraction and acid precipitation [16]. Walnut powder was defatted with n-hexane and sieved with an 80 mesh sieve. The defatted walnut flour was adjusted to pH 9.0 with 1 mol/L NaOH, and after stirring for 3 h, centrifuged at 8000× *g* for 15 min. The supernatant was adjusted to pH 4.5 with 1 mol/L HCl and centrifuged again at 8000× *g* for 15 min. The pH of the precipitate was adjusted to 7.0, after dialyzing for 3 days using ultrapure water. Walnut protein isolate (WPI) precipitates were obtained by freeze-drying and stored at −20 °C for subsequent analysis.

### 2.3. Analysis of Amino Acid Composition

The composition and content of amino acids were analyzed by HPLC (Hitachi Amino Acid Analyzer, Tokyo, Japan). The amino acid composition was analyzed with reference to this method [17]. The result of amino acid composition was expressed in grams per 100 grams of protein.

### 2.4. Measurement of Protein Carbonyl Groups

Protein carbonyl content was evaluated using the DNPH method [18]. Carbonyl content was calculated using a molecular absorbance coefficient of 22,000 M^−1^cm^−1^ at 370 nm and described as nmol/mg protein. 

### 2.5. Dityrosine Content Measurement

The content of dityrosine in WPI was determined with reference to this method [19]. Notably, 20 mg of WPI samples were dispersed in 20 mL of 0.02 mol/L phosphate buffer (0.6 mol/L KCl, pH 6.0). The content of dityrosine in the filtrate was determined using a fluorescence spectrophotometer with an emission wavelength of 420 nm and an excitation wavelength of 325 nm. 

### 2.6. Determination of Free Sulfhydryl Groups

The free sulfhydryl group (-SH) in WPI was determined by the method of Li et al. with slight modification [13]. Notably, 15 mg of WPI samples were dissolved in 5.0 mL of pH 8.0 Tris-Gly buffer. After stirring for 30 min, the solution was centrifuged at 1000× *g* for 15 min. The concentration of walnut proteins in the supernatant solution was calculated using the biuret method. Notably, 5 mL of supernatant was reacted with 0.05 mL of DTNB reagent for 1 h and protected from light. A UV-visible spectrophotometer was used to measure the absorbance values at 412 nm, and the sulfhydryl content was calculated using a molar extinction coefficient of 13,600 M^−1^cm^−1^.

### 2.7. Determination of Protein Solubility

The solubility of WPI was measured by the biuret method [20]. Notably, 0.05 g WPI sample was dissolved in 10 mL deionized water and stirred for 2 h. Then, the solution was centrifuged at 5000 rpm for 15 min at 4 °C. The solubility of WPI was expressed as the percentage of protein concentration after centrifugation to the protein concentration before centrifugation. 

### 2.8. Characterization of the Secondary Structure—FT-IR

The secondary structure of WPI was determined by using the method of Wu et al. [21]. The FT-IR spectra of WPI samples in the wavelength range from 4000–400 cm^−1^ was measured. The secondary structures of the protein were exhibited in the range of 1600–1700 cm^−1^, and curve fitting was performed by Peak-Fit version 4.04 software to evaluate the percentage of secondary structures.

### 2.9. Measurement of Intrinsic Fluorescence

The intrinsic fluorescence spectra of WPI were evaluated in a previous study [13]. The WPI samples were dissolved in 0.01 mol/L of pH 7.0 phosphate buffer solution. After stirring for 2 h, the solution was centrifuged at 5000 rpm for 15 min and adjusted the supernatant concentration to 0.1 mg/mL. Intrinsic fluorescence spectra were determined at an excitation wavelength of 280 nm and the emission spectra were scanned in the range of 280–460 nm.

### 2.10. Surface Hydrophobicity (H_0_) Measurement

Surface hydrophobicity of WPI was determined by ANS (1-anilinaphthalene-8-sulfonic acid) [22]. WPI samples (mass concentration range: 0.005–0.5 mg/mL) were scattered in 0.01 mol/L of pH 7.0 phosphate buffer. After the reaction of 3 mL of protein solution with 30 µL of 8 mmol/L ANS solution, the fluorescence intensity was measured under fluorescence conditions with excitation wavelength of 385 nm and emission wavelength of 490 nm, the slope of fluorescence intensity versus protein concentration was used to characterize the surface hydrophobicity.

### 2.11. Particle Size Determination

The particle size was determined by dynamic light scattering. WPI samples were dissolved in 0.01 mol/L of pH 7.4 phosphate buffer and stirred for 2 h. Subsequently, the solution was centrifuged at 8000× *g* for 10 min and protein concentration was adjusted to 0.1 mg/mL for determination.

### 2.12. SDS-PAGE of WPI

Protein oxidation in walnuts under storage conditions was assessed by SDS-PAGE [23]. Notably, 2 mg/mL WPI samples were mixed with 2× loading buffer and then heated at 100 °C for 5 min. Notably, 10 μL of the prepared samples were added to the gels (consisting of 5% polyacrylamide concentrate and 12% polyacrylamide separation gels). Protein subunit separation was performed using a Mini-PROTEAN electrophoresis system (Bio-Rad Laboratories, Hercules, CA, USA) at 80 and 120 V. The prepared gel samples were stained with 0.25% Coomassie Brilliant Blue R-250 for 20 min and then decolorized with a mixture solution of 7.5% acetic acid and 25% methanol. Afterward, gel images were obtained by an imaging system (Bio-Rad Laboratories, Hercules, CA, USA).

### 2.13. Determination of Protein Molecular Weight Distribution

The molecular weight distribution of WPI during storage was determined using LC-2010A liquid chromatogram (Shimadzu, Kyoto, Japan) with a UV detector and a column of Shodex PROTEIN KW-804 (8 mm × 300 mm, Tosoh Biosep, Tokyo, Japan). Notably, 1 mg/mL protein solution was prepared with 0.05 mol/L phosphate buffer (pH 7.0, containing 0.2 mol/L Na_2_SO_4_), and filtered with 0.45 μm microporous filter membrane. Notably, 10 μL of protein solution was collected for examination. The eluate was measured at 280 nm using a mobile phase of 0.05 mol/L of pH 7.0 phosphate buffer (0.2 mol/L Na_2_SO_4_) with 0.8 mL/min flow rate.

### 2.14. In Vitro Digestion of WPI

Simulation of gastric and intestinal digestion of WPI with reference to the protocol of static in vitro digestion [24]. Notably, 20 mg/mL of walnut protein solution was adjusted to pH 2.0 with 1.0 mol/L HCl, followed by adding pepsin (2000 U/mg, based on protein) and shaking in a water bath shaker at 37 for 1 h. Subsequently, the solution was removed and the gastric digest was adjusted to pH 7.0 with 1.0 mol/L NaOH, added trypsin (250 U/mg, based on protein), and was continuously shaken for 2 h at 37 °C in a water bath shaker. Afterward, the gastrointestinal digest fluid was placed in a boiling water bath for 5 min to stop digestion, and after cooling, the digest fluid was centrifuged at 10,000× *g* for 10 min and stored at −20 °C for further use.

### 2.15. Degree of Hydrolysis (DH) Measurement

The digestibility of proteins is usually expressed using the degree of hydrolysis, and the OPA method was used to analyze the hydrolysis of WPI digestion [25]. Notably, 40 mg/mL ortho-phthalaldehyde reagent (dissolved in methanol) was prepared, followed by 2.5 mL sodium dodecyl sulfate (SDS), and 100 μL β-mercaptoethanol was added successively with a fixed volume of 50 mL. Notably, 200 μL walnut protein solution was added to 4 mL OPA reagent and reacted for 2 min at 35 °C. Subsequently, the absorbance values of protein samples were measured at 340 nm. The degree of hydrolysis (DH) of WPI was calculated as follows Equations (1) and (2):

(1) $$\mathrm{DH}=h/h_{tot}\times 100\%$$

(2) $$h=\left(serineNH_{2}-\beta \right)/\alpha$$

where *β* is 0.342 mequv/g, α is 0.970 mequv/g, *h_tot_* is the constant of WPI 7.8 mequv/g.

### 2.16. Molecular Weight Distribution of Peptide Determination

Peptide molecular weight distribution was determined by referring to this method of Yu et al. with slight modifications [26]. Notably, 10 μL protein digestive solution (1 mg/mL) was taken for analysis, using LC-2010A HT liquid chromatography (equipped with UV detector) and a column of TSKgel 2000 SWXL 300 × 7.8 mm. Then, the digest was detected at 214 nm using acetonitrile/water/trifluoroacetic acid (45/55/0.1, *V*/*V*) as the mobile phase with the flow rate of 0.5 mL/min. Standards for the molecular weight correction curve were Cytochrome C (MW12327); Aprotinin (MW 6511.433); Bacitracin (MW1421.69); Eth-Eth-Try-Arg (MW 451.2) and Gly-gly-gly (MW 189.17). The lg Mw to elution time of the standards was used as the standard curve (Y = −0.2179X + 6.6516, R^2^ = 0.9973).

### 2.17. Data Analysis

The data were analyzed using SPSS Statistics for Windows version 17.0, (2008; SPSS Inc., Chicago, IL, USA). One-way analysis of variance (ANOVA) was used to compare between and within group differences, followed by Duncan’s test, and significant differences were considered as *p* < 0.05. Data were expressed as the mean standard deviation of at least two replicate samples. Graphical plotting was performed using Origin 2021 software (OriginLab Corporation, Northampton, MA, USA). Pearson correlation analysis was used to explore the relationship between walnut protein quality indicators and digestive characteristics in vitro during storage.

## 3. Results

### 3.1. Amino Acid Composition Analysis in Different Packaging Strategies

**Figure 1:**
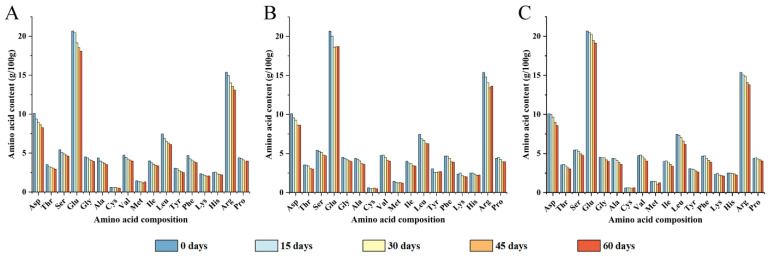
Amino acid composition analysis of walnut kernels with different packaging materials at 0, 15, 30, 45, and 60 days of storage ((**A**) PE-sealed packaging; (**B**) PA/PE vacuum packaging; (**C**) PET/AL/PE vacuum packaging).

Walnut protein usually contains 18 kinds of amino acids. Yet tryptophan was destroyed completely in the determination process, so it was not analyzed. As shown in Figure 1, the contents of 17 types of amino acids were roughly the same in different packaging, with glutamic acid having the highest content, at 20.84%, followed by arginine and aspartic acid, at 15.50% and 10.18%, respectively. All amino acid contents decreased during storage. After 60 days of storage, the content of total amino acid in PE-sealed packaging decreased significantly, followed by PA/PE and PET/AL/PE, respectively. Specifically, the contents of amino acids such as Ala, Phe, Leu, Cys, Val, Ile, and Asp decreased by more than 15% in all three packages, in addition to the significant decreases shown by Thr, Ser, and Tyr in PE-sealed bags. Previous research has found similar results [27]. Environmental factors, especially oxygen concentration, tend to cause oxidative rancidity of walnuts, resulting in reactive free radicals and lipid secondary oxidation products that lead to amino acid modification into multiple forms of derivatives (e.g., carbonyl derivatives, α-ketoacyl derivatives, amide derivatives, etc.) [28]. In addition, the active side chains of amino acids containing sulfur, amino, imidazole, and indole groups are particularly susceptible to oxidation by lipids and their derivatives, such as the sulfur-containing amino acid Met, which is susceptible to oxidation to methionine sulfoxide derivatives [29]. The essential and hydrophobic amino acids were particularly sensitive to oxidation and noticeably decreased during storage (Table 1). 

### 3.2. Oxidation of WPI in Different Packaging Strategies

Oxidative stress in food storage and processing causes protein oxidation, which alters the side chains of amino acids and the peptide backbone. These modifications are often followed by alterations in protein structure, such as the formation of carbonyl derivatives, loss of sulfhydryl groups, modification of aromatic amino acids, and generation of protein covalent crosslinks [30].

**Figure 2:**
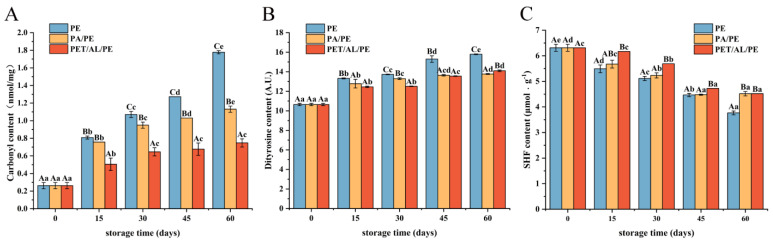
Influence of storage conditions on the content of carbonyl (**A**), dityrosine (**B**), and free sulfhydryl (**C**). In the figures, capital letters represent the differences among package materials in each storage period, lower letters show differences among storage periods in each package at *p* < 0.05 error level according to Duncan’s multiple range test. The error bar represents the standard error of the mean value.

Figure 2A–C showed the indicators related to protein oxidation. The non-enzymatic irreversible process of carbonylation is associated with the oxidative stress of proteins, and carbonyl groups are often considered an important indicator of the degree of protein oxidation [31]. The change in carbonyl content was shown in Figure 2A, where the content of carbonyl compounds increased with the increase in storage time. After 60 d of storage, the carbonyl content of WPI in PE-sealed packaging increased significantly from 0.26 nmol/mg to 1.78 nmol/mg (*p* < 0.05). In contrast, the PET/AL/PE vacuum packaging showed a slower change. Previous studies have shown that the free catalytic release of iron ions and the cleavage of hydroperoxides led to protein oxidation and the formation of protein carbonyl compounds under the influence of environmental factors [32]. Lipid peroxidation generates lipid radicals and reactive aldehydes, which encouraged the production of protein peroxyls by targeting protein backbones and side chains of amino acids, thus increasing the protein carbonyl content [33]. Furthermore, secondary products of lipid oxidation such as malondialdehyde could interact with the nucleophilic side chains of lysine, cysteine, and histidine residues, leading to the formation of protein carbonyls [34]. The findings revealed that free radicals, lipid peroxidation radicals, and secondary oxidation products caused cross-linking cleavage and modification of protein α-carbon atoms and amino acid side chains (e.g., Cys, Met, Val, Lys, Arg, His, Tyr, Pro residues) into carbonyl compounds [33]. Whereas low-oxygen and light-blocking packaging materials such as PET/AL/PE vacuum packaging could effectively prevent protein oxidation.

The change of dityrosine content has also been used to indicate protein oxidation degree. As shown in Figure 2B, dityrosine content increased with storage time. The change in tyrosine content was slower in vacuum packaging than in non-vacuum packaging, and the difference in dityrosine content between PA/PE and PET/AL/PE vacuum packaging for 45 d was not significant (*p* < 0.05). The results showed that protein oxidation occurred and tyrosine residues were oxidized to form dityrosine. However, vacuum packaging could slow protein oxidation.

Cys residue is the most sensitive amino acid residue of all amino acids, and it is more susceptible to oxidative attack, leading to a change in protein sulfhydryl content. As shown in Figure 2C, free sulfhydryl content in PE-sealed packaging decreased significantly from 6.4 μmol/g to 4.0 μmol/g at 60 days of storage (*p* < 0.05). With a slower decrease under vacuum conditions, especially for PET/AL/PE vacuum packaging. The results showed that the walnut protein structure was disrupted during storage, exposing more reactive groups (mainly hydrophobic groups and cysteine) and that cysteine and methionine residues were easily oxidized to the thiol group [35], which also corresponded to the reduction of sulfur-containing amino acids in Figure 1. Meanwhile, hydroxyl radicals and lipid peroxidation were generated during storage, which readily attacked the free thiol groups, forming disulfide bonds and a reversible form of sulfinic acid or irreversible form of sulfinic acid [33,36]. The increase in carbonyl and dityrosine content during storage, which indicated denaturation, intermolecular disulfide bond cross-linking, or the oxidation of protein, would be confirmed in subsequent studies. 

### 3.3. Characterization of Protein Structure in Different Packaging Strategies

#### 3.3.1. Secondary Structure (FT-IR) Analysis

**Figure 3:**
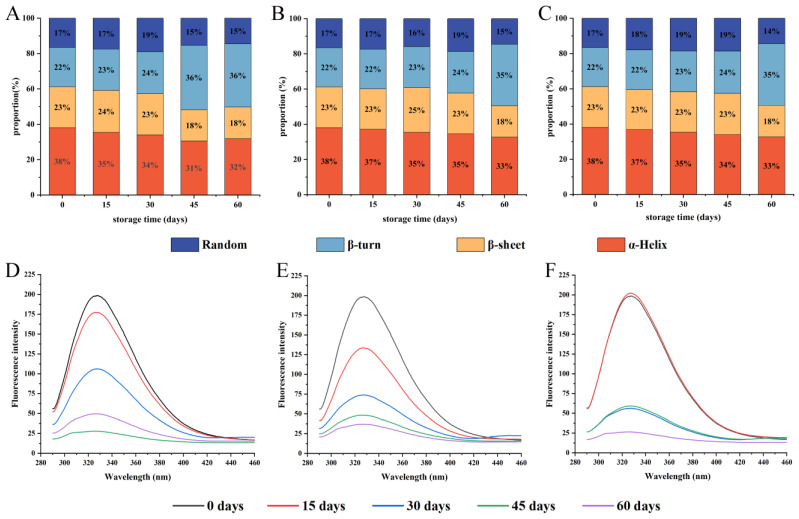
Effects of packaging materials on secondary structure (**A**–**C**) and endogenous fluorescence intensity (**D**–**F**) of walnut protein at 0, 15, 30, 45, and 60 days of storage. (**A**,**D**): PE-sealed packaging; (**B**,**E**): PA/PE vacuum packaging; (**C**,**F**) PET/AL/PE vacuum packaging.

The structure of proteins in different states can be measured using FT-IR. The change of protein secondary structure is reflected by the amide I band (wavenumber range of 1700–1600 cm^−1^) [37]. Figure 3A–C showed the percentage of protein secondary structures in WPI. In particular, the α-helix is a dense and ordered structure that provides stable energy mainly through hydrogen bonding, which is important for the formation of protein structures. As the storage time prolonged, the percentage of α-helix and β-sheet decreased, while the percentage of β-turn and random coil increased, which was consistent with the changes in rice protein secondary structure during storage, indicating that protein oxidation caused the protein secondary structure to become disordered and loose [38]. The secondary structure of WPI changed slowly under vacuum conditions. The difference between PA/PE and PE/PET/AL vacuum packaging was not significant (*p* < 0.05). After 60 days, the β-sheet decreased significantly and β-turn increased significantly, and the percentage change of the β-sheet was significantly higher than that of the α-helix. Some studies have shown that a similar phenomenon was found, pointing out that the β-sheet was more sensitive to the change in environmental conditions compared to other secondary structures [39]. In this study, storage conditions facilitated protein oxidation, causing the unfolding in α-helix and a decrease in the ordered structure. The increase in protein molecular disorder exposed surface hydrophobic residues that could lead to increased surface hydrophobicity and decreased protein solubility and digestibility [22]. It has been shown that the secondary structure of proteins also correlates with their digestive properties and nutritional value, such as α-helix content, which was positively correlated with protein digestibility [40]. These discoveries would be confirmed in subsequent studies.

#### 3.3.2. Intrinsic Fluorescence Analysis

The changes in the protein tertiary structure were commonly estimated by intrinsic fluorescence, which also could reflect the change of the tryptophan residues microenvironment [41]. As shown in Figure 3D–F, native walnut protein samples exhibited a high fluorescence intensity with a peak at 328 nm. With the extension of storage time, the peak position of the intrinsic fluorescence spectral gradually shifted blue, from 328 to 324.1 nm for PE-sealed packaging, from 328 to 327.0 nm for PA/PE vacuum packaging, and from 328 to 326.9 nm for PET/AL/PE vacuum packaging. Tryptophan is sensitive to environmental factors due to the lowest single-electron oxidation potential. As storage time increased, tryptophan residues were converted into unstable tryptophan radicals by radicals dehydrogenation reactions and combined with split oxygen atoms to form tryptophan peroxide radicals, which were eventually transformed to kynurenine, leading to the decrease of intrinsic fluorescence intensity [42]. In addition, the formation of protein aggregates masking tryptophan residues also results in a decrease in fluorescence intensity [43]. Tryptophan fluorescence quenching is a manifestation of protein oxidation [33]. At the same time, the blue-shift of peak position with the prolonged storage time of walnuts showed that the cross-linking of protein oxidation modification led to the shift of the tryptophan residue microenvironment from polar to non-polar [44].

#### 3.3.3. Surface Hydrophobicity (H_0_) Measurement

**Figure 4:**
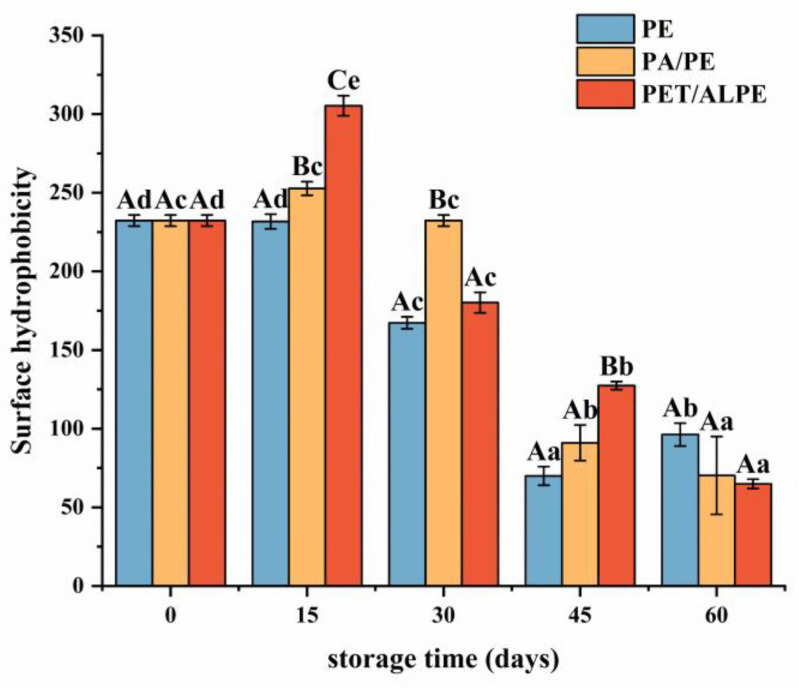
Effects of packaging materials on surface hydrophobicity of walnut protein at 0, 15, 30, 45, and 60 days of storage. In the figure, capital letters represent the differences among package materials in each storage period, lower letters show differences among storage periods in each package at *p* < 0.05 error level according to Duncan’s multiple range test. The error bar represents the standard error of the mean value.

Surface hydrophobicity is tightly linked to the functional characteristics and structural stability of proteins [45]. As depicted in Figure 4, the surface hydrophobicity of WPI increased at the beginning of storage and gradually decreased after 15 days of storage. WPI is a globular protein wrapped by hydrophilic groups and hydrophobic groups, which is susceptible to environmental conditions (e.g., temperature, oxygen, humidity, light, etc.), leading to protein structure damage and exposure of hydrophobic groups.

Meanwhile, lipid oxidation caused conformational changes in proteins that resulted in protein extension and unfolding during storage. Structural modifications resulted in the exposure of side chain groups of aromatic and hydrophobic aliphatic amino acids wrapped inside of protein, thus increasing the surface hydrophobicity of WPI [46]. Furthermore, the dissociation of protein subunits and unfolding of subunit polypeptide chains may increase surface hydrophobicity [47]. However, after storage for 15 days, the surface hydrophobicity decreased, which could be attributed to the oxidative aggregation of protein molecules through hydrophobic interactions as the increase in oxidation degree, as well as covalent structural modification of exposed hydrophobic amino acid residues (e.g., tryptophan residues), and structural changes of oxidized surface hydrophilic amino acids to form partially hydrophilic groups (e.g., protein carbonyl groups) [48].

### 3.4. Aggregation of Walnut Protein Isolates during Storage

#### 3.4.1. Solubility and Particle Size Analysis

**Figure 5:**
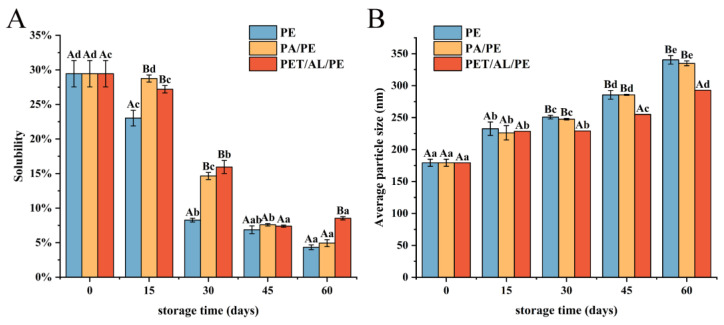
Effects of packaging materials on solubility (**A**) and particle size (**B**) of walnut protein isolate. In the figures, capital letters represent the differences among package materials in each storage period, lower letters show differences among storage periods in each package at *p* < 0.05 error level according to Duncan’s multiple range test. The error bar represents the standard error of the mean value.

Protein solubility is an index to further evaluate protein aggregation degree by determining the concentration of insoluble molecules in protein solution [35]. As shown in Figure 5A, protein solubility gradually decreased during storage due to slow oxidation, which resulted in insoluble aggregates of high molecular weight formed by increased disulfide bond formation and hydrophobic interactions [49]. PE-sealed packaging showed the fastest decrease in solubility. After storage for 45 days, there was no significant difference in the solubility of WPI between the three packaging methods (*p* < 0.05).

Particle size reflects protein aggregation and dispersion to some extent. Dynamic light scattering (Laser particle size analyzer) is usually applied to detect the creation of protein aggregates. As seen in Figure 5B, the average particle size of WPI increased with storage time due to protein oxidative aggregation. As well, solubility was negatively correlated with the particle size. The particle size of PET/AL/PE vacuum packaging increased more slowly than the other two packaging methods, which indicated that PET/AL/PE vacuum packaging could better prevent protein oxidation and was beneficial for the long-term storage of walnuts.

#### 3.4.2. SDS-PAGE

**Figure 6:**
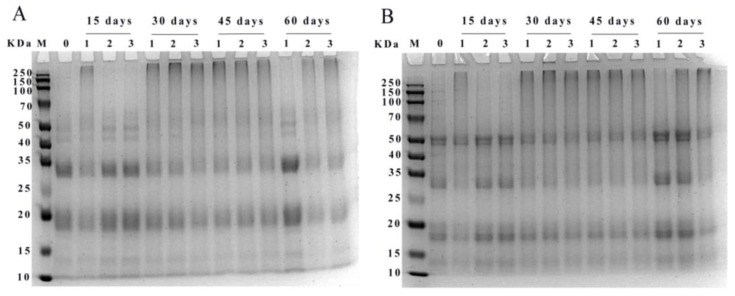
SDS-PAGE pattern of WPI under different storage conditions ((**A**) reducing; (**B**) non-reducing; 0: storage for 0 days; 1: PE-sealed packaging; 2: PA/PE vacuum packaging; 3: PE/PET/AL vacuum packaging).

The reducing (A) and non-reducing SDS-PAGE (B) electropherograms of WPI was shown in Figure 6. Protein subunits of walnut were mainly distributed at 10–15, 15–20, 25–35, and 40–50 KDa. The major protein bands at 15–20 and 25–35 KDa became shallower with increasing storage time. After storage for 15 days, high molecular weight protein aggregates appeared at the top gels in PE-sealed packaging, and the bands of WPI in the range of 40–50 KDa were slightly deepened in PA/PE and PET/AL/PE vacuum packages. After 30 days of storage, the WPI samples in different packages appeared as aggregates at the top of the strip. This may be due to the oxidation of proteins by free radicals generated during storage, leading to covalent cross-linking and the formation of high molecular weight aggregates of WPI. However, after 60 days of storage, the high molecular weight protein bands in the accumulation layer became shallower or disappeared, indicating that the deepening of protein oxidation also led to the degradation of WPI, producing discrete bands in the low molecular weight region. Similar changes were found during storage at 30 °C, where the high molecular protein subunits of rice were gradually broken down into lower molecular proteins (wheat gliadin, albumin, α-globulin, and glutenin) [38]. Furthermore, bands in the non-reducing mode were deeper than in the reducing mode, indicating that disulfide bonds were involved in the oxidative cross-linking reaction.

#### 3.4.3. Molecular Weight Distribution of WPI Analysis

**Figure 7:**
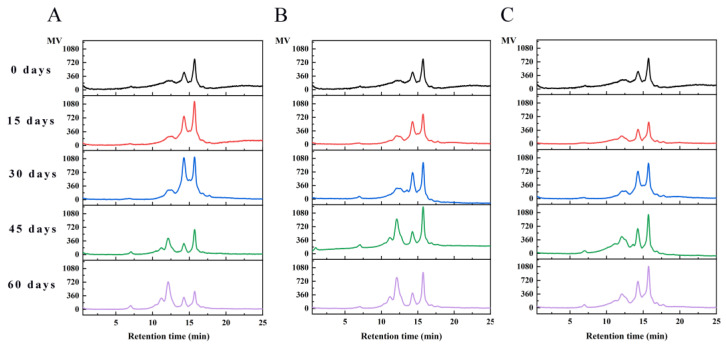
Molecular weight distribution analysis of walnut protein isolate under different storage conditions ((**A**) PE-sealed packaging; (**B**) PA/PE vacuum packaging; (**C**) PE/PET/AL vacuum packaging).

HPLC is an effective method for evaluating protein aggregation and can further analyze the distribution of walnut protein molecular weight. The molecular weight distribution of WPI was given in Figure 7. The percentage of peak areas of protein molecular weight distribution was shown in Table 2. Native walnut protein isolates exhibited two peaks at retention times of 14.25 and 15.72 min. After 30 days of storage, the protein molecular weight distribution added a new peak at 12.11 min in PE and PA/PE. As the storage time progressed, five elution peaks were fully separated, and the corresponding retention times were 7.00, 11.16, 12.11, 14.25, and 15.72 min, respectively. Peaks with retention times of 7.00 min had molecular weight distribution ranging from 200 to 2000 KDa. Peaks with molecular weights of 100–200 KDa, 50–100 KDa, 10–50 KDa, and 3–10 KDa appeared at 11.16, 12.11, 14.25, and 15.72 min, respectively. The main molecular weight distribution of walnut proteins is in the range of 3–50 KDa. With the increase in storage time, the percentage of peak area gradually increased at 11.16, 12.11, and 7.00 min and decreased at 14.25 and 15.72 min. The appearance of new peaks at shorter storage times corresponded to walnut protein aggregates with higher molecular weights. The results suggested that the proteins of walnut were oxidized and produced protein aggregates, as also reported for rice and peanut proteins oxidized by malondialdehyde and peroxyl radicals [18,50].

### 3.5. Properties of In Vitro Digestion in Different Packaging Strategies

#### 3.5.1. Degree of Hydrolysis Analysis

**Figure 8:**
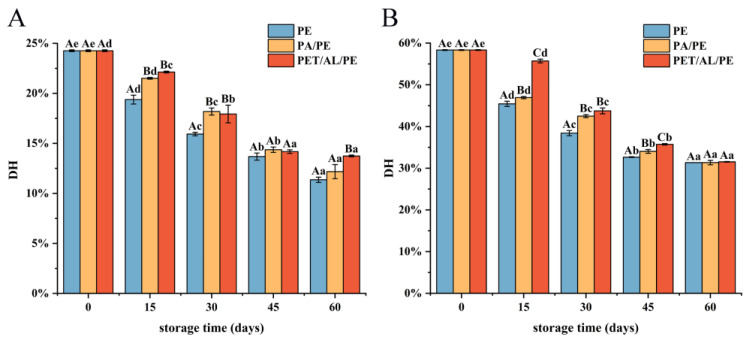
Effect of storage conditions on gastrointestinal digestibility of walnut protein isolate ((**A**) degree of hydrolysis after 2 h of gastric digestion; (**B**) Degree of hydrolysis after 2 h intestinal digestion). In the figure, capital letters represent the differences among package materials in each storage period, lower letters show differences among storage periods in each package at *p* < 0.05 error level according to Duncan’s multiple range test. The error bar represents the standard error of the mean value.

DH% indicates the percentage of peptide bond breakage. Figure 8 showed the degree of hydrolysis of WPI in gastric digestion (A) and intestinal digestion (B). The degree of hydrolysis decreased with increasing storage time. During the gastric digestion phase, the greatest decrease in DH% was observed for PE-sealed packaging, from 24.26% to 11.36%, while the difference in DH% between PA/PE and PET/AL/PE vacuum packaging was not significant at 15, 30, and 45 days. During intestinal digestion, DH% decreased significantly from 58.33% to 31.34%. After storage for 60 days, the degree of hydrolysis did not differ significantly for the three packages (*p* < 0.05). The decrease in DH% was mainly due to protein oxidative aggregation. As a result of the cross-linking, some protease cleavage sites were modified or masked, significantly reducing the digestibility in vitro. This result corresponded with earlier studies [51,52]. Changes in protein secondary structure have an impact on the recognition of gastrointestinal digestive enzymes, and the β-sheet content has been reported to be associated with the formation of a dense and regular structure of the protein [53]. Therefore, the stable conformation somewhat limited the access to proteases. Furthermore, several studies have indicated that the digestive properties and nutritional value of protein depend to a large extent on the amino acid composition. The amino acid composition of walnut protein was similar to that of cashew protein, with the highest content of glutamic acid and arginine, so there is a high in vitro digestibility. Pepsin cleaved specifically peptide bonds with aromatic amino acids (e.g., Phe, Tyr, and Trp) or Leu at the amino and carboxyl terminus. Trypsin specifically cut carboxyl-terminal peptide bonds of the basic amino acid (Arg and Lys), whereas chymotrypsin specializes in breaking the peptide bonds of aromatic amino acids or hydrophobic amino acid residues. Therefore, the high digestibility of nut proteins could be related to the high content of acidic amino acids, hydrophobic amino acids, and arginine in the protein [54].

#### 3.5.2. Characterization of Peptides Molecular Weight Distribution

**Figure 9:**
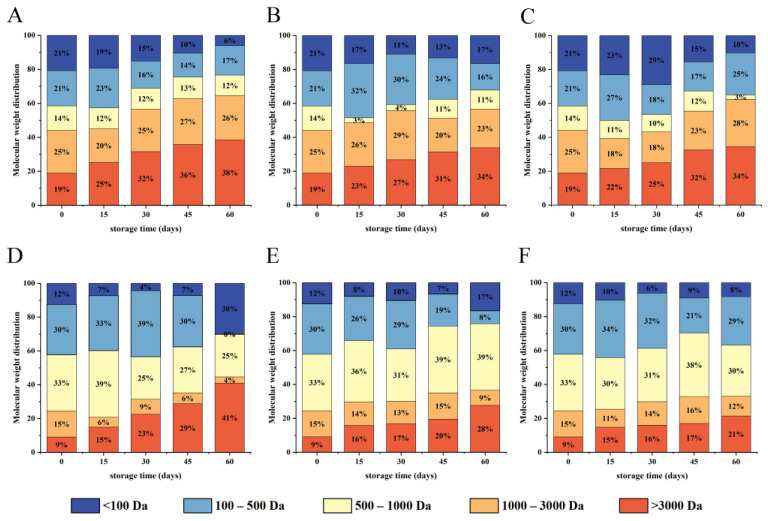
The molecular weight distribution of WPI under different storage conditions ((**A**,**D**) PE; (**B**,**E**) PA/PE; (**C**,**F**) PET/AL/PE) after in vitro pepsin digestion (**A**–**C**) and trypsin digestion (**D**–**F**).

Peptide molecular weight distribution was shown in Figure 9. Before digestion, the molecular weight of WPI was primarily distributed between 3–11.5 KDa, with the peptides (<3000 Da) not detected. As shown in Figure 9A–C, the high molecular weight subunits (>3000 Da) were gradually decomposed, and low molecular weight oligopeptides were slowly increased after pepsin digestion. Peptides with molecular weights ranging from 1000 to 3000 Da were the main components of gastric digestive juices. The finding confirmed that pepsin is an endopeptidase with a highly specific restriction site that causes the protein to form large peptide fragments [55]. Although pepsin could not completely digest walnut protein, it could change the natural conformation of WPI and promote further digestion of trypsin.

The molecular weight distribution of peptide after trypsin digestion was displayed in Figure 9D–F. The results showed that walnut protein of different packaging materials could be digested into oligopeptides and amino acids with lower molecular weight by trypsin. The component with a molecular weight of less than 3000 Da accounted for 91% of the gastrointestinal digestive fluid in the native protein. Among components with molecular weights of 100–500 Da and 500–1000 Da, accounted for 30% and 33% of gastrointestinal digestive fluid, respectively, and were the main components. After pepsin and trypsin hydrolysis, the content of low molecular weight peptides (<3000 Da) decreased gradually as storage time increased. Because oxidation caused protein cross-linking and aggregation, as well as masking the protease cleavage sites, protein digestibility decreased [56]. Simultaneously, studies revealed that peptides of low molecular weight with 2–10 amino acid residues had high antioxidant activity [57]. However, WPI underwent oxidative aggregation during storage, which blocked the cleavage site of protease and inhibited the production of antioxidant-active peptides. After 60 days, the molecular weights of <3000 Da with vacuum packaging were significantly higher than with non-vacuum packaging, especially PET/AL/PE vacuum packaging, indicating that walnut kernels with PET/AL/PE vacuum packaging had the best digestibility and that PET/AL/PE vacuum packaging had a positive effect on the regulation of walnut quality.

### 3.6. Pearson Correlation

**Figure 10:**
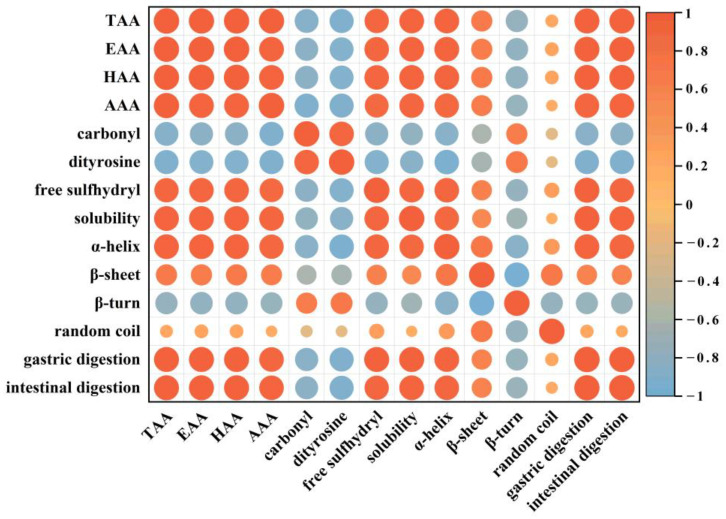
Pearson correlation of the different indexes in walnut kernels with different packaging, which were stored at 40 °C, 70% relative humidity for 0, 15, 30, 45, and 60 days. In the figure, the color gradation and circle size correspond to the correlation coefficient.

Pearson correlation analysis was used to explore the relationship between protein quality and in vitro digestibility of walnut during storage. Figure 10 depicted the correlation among amino acids, oxidation indexes, and structural and in vitro digestibility of walnut protein. The findings revealed that amino acid was negatively correlated with carbonyl and dityrosine (r = −0.88, r = −0.91; *p* < 0.05), but positively correlated with in vitro digestibility in the gastric and intestinal phases (r = 0.97, r = 0.97; *p* < 0.05), indicating that oxidation caused amino acid modifications, and some amino acid residues were oxidatively modified to carbonyl derivatives (e.g., Cys, Met, Val, Lys, Arg, His, Tyr, Pro, etc.) and dityrosines (e.g., tyr) (Figure 2A,B).

Protein oxidation indicators such as carbonyl and dimeric tyrosine content were negatively correlated with the degree of hydrolysis in both gastric and intestinal digestion (r = −0.84, r = −0.83; *p* < 0.05 and r = −0.91, r = −0.91; *p* < 0.05, respectively). It was shown that the degradation of amino acids and the formation of protein oxidative aggregates during storage significantly reduced the solubility of WPI (Figure 5A) and the in vitro digestibility during the gastrointestinal phase (Figure 8A,B).

In addition, changes in the secondary structure such as α-helix and β-sheet content were positively correlated with protein digestibility in both gastric and intestinal phases (r = 0.95, r = 0.94; *p* < 0.05 and r = 0.64, r = 0.62; *p* < 0.05). α-helix structure played a major role in maintaining the structural stability of the protein, mainly through hydrogen bonding. β-sheet was also associated with the dense and ordered structure of the protein [58]. However, with the increase in storage time, the protein structure undergoes oxidative modification and the recognition site of protease is changed, which decreases the hydrolysis sensitivity of protease and leads to a decrease in digestibility. Previous studies have reported that protein digestibility cannot be influenced by a single factor [59]. Therefore, in the study, we have found that degradation of amino acids, formation of protein aggregates, and structural changes during storage combined to cause a decrease in the in vitro digestibility of walnut proteins.

## 4. Conclusions

With increasing storage time, the quality, structure, and digestibility characteristics of walnut protein in three different packages changed to different degrees with the extension of storage time, and the vacuum degree had a significant effect on the quality of walnut protein. During storage, the carbonyl and dityrosine content increased significantly and the free sulfhydryl content decreased significantly. Particularly, significant change in carbonyl value from 0.26 nmol/mg to 1.78 nmol/mg for PE-sealed packaging. Oxidation causes amino acid modifications and a significant reduction of essential amino acid content. After storage for 6 months, the secondary structure of walnut proteins in PE-sealed packaging changed significantly, α-helix structure decreased from 38.03% to 31.82% and β-turn increased from 22.22% to 36.44%, the protein structure tended to be disordered, and loose. Disruption of walnut protein structure during storage, exposing more hydrophobic groups. The surface hydrophobicity first increased and then decreased, indicating that protein was unfolded at the beginning of storage. After storage for 15 days, protein oxidation deepened and oxidative aggregation occurred, resulting in poor dispersion of WPI. It was also demonstrated in SDS-PAGE and protein molecular weight distribution maps that the proteins formed large molecular weight aggregates after 15 days of storage. Pearson correlation analysis showed that degradation of amino acids, formation of protein aggregates, and changes in protein structure during storage led to masking of the active sites of proteases and reduced the digestibility of WPI. PET/AL/PE vacuum could more effectively inhibit the oxidation of walnut proteins, prolong the storage period, and reduce the nutritional losses of walnuts. Therefore, this study can be used as a theoretical reference for effective walnut storage and to better understand how the quality and nutritional properties of nuts rich in lipid and protein change during storage and processing. On this basis, the mechanism and regulatory strategies of protein quality changes in walnut will be investigated further in order to better promote the development and application of nuts rich in lipids and proteins.

## Figures and Tables

**Table 1:** Amino acid composition analysis of walnut kernels with different packaging materials at 0, 15, 30, 45, and 60 days of storage (TAA is a total amino acid, EAA is an essential amino acid, HAA is a hydrophobic amino acid, and AAA is acidic amino acids).

Storage Time (Days)	Packaging Methods	Amino Acid Content (g/100 g)			
		TAA	EAA	HAA	AAA
0	—	99.21	30.69	31.06	30.77
	PE	94.78	28.72	28.94	29.85
15	PA/PE	95.83	29.92	30.19	29.58
	PET/AL/PE	98.90	30.97	31.18	30.49
	PE	89.81	27.31	27.65	28.10
30	PA/PE	91.04	28.39	28.76	27.86
	PET/AL/PE	95.79	29.35	29.66	29.88
	PE	86.37	26.18	26.40	27.17
45	PA/PE	86.87	26.43	26.73	27.31
	PET/AL/PE	90.45	27.45	27.74	28.44
	PE	84.21	25.71	26.03	26.35
60	PA/PE	86.40	26.03	26.33	27.30
	PET/AL/PE	87.28	26.09	26.37	27.70

**Table 2:** Percentage of peak area of molecular weight distribution of walnut protein isolate under different storage conditions.

Storage Time/Days	Packaging Methods	Percentage of Peak Area (%) and Its Corresponding Retention Time				
		7.00/min >1000 KDa	11.16/min 105.93 KDa	12.11/min 53.44 KDa	14.25/min 11.49 KDa	15.72/min 3.97 KDa
0		—	—	—	39.27	60.73
	PE	—	—	—	45.50	54.50
15	PA/PE	—	—	—	45.24	54.76
	PE/PET/AL	—	—	—	32.53	67.47
	PE	—	—	2.46	55.72	41.82
30	PA/PE	1.60	—	—	38.43	59.97
	PE/PET/AL	—	—	—	47.01	52.99
	PE	2.14	3.71	36.15	16.53	43.61
45	PA/PE	2.21	6.85	41.52	13.37	36.05
	PE/PET/AL	—	—	17.85	29.62	52.53
	PE	3.67	12.04	50.40	12.58	21.32
60	PA/PE	—	7.74	45.01	13.93	33.33
	PE/PET/AL	2.74	—	5.61	37.14	54.51

## Data Availability

The data presented in this study are available on request from the corresponding author.
